# Monitoring of the Biotechnological Production of Dihydroxyacetone Using a Low‐Field ^1^H NMR Spectrometer

**DOI:** 10.1002/bit.28901

**Published:** 2024-12-10

**Authors:** Lukas Mahler, Ebru Tasdemir, Anna Nickisch‐Hartfiel, Christian Mayer, Martin Jaeger

**Affiliations:** ^1^ Department of Physical Chemistry University Duisburg‐Essen Essen North Rhine‐Westphalia Germany; ^2^ Department of Chemistry and ILOC Niederrhein University of Applied Sciences Krefeld North Rhine‐Westphalia Germany

**Keywords:** biocatalysis, online NMR, platform chemicals, process analytical technologies, real‐time monitoring

## Abstract

The concept of sustainable production necessitates the utilization of waste and by‐products as raw materials, the implementation of biotechnological processes, and the introduction of automated real‐time monitoring for efficient use of resources. One example is the biocatalyzed conversion of the reusable by‐product glycerin by acetic acid bacteria to dihydroxyacetone (DHA), which is of great importance to the cosmetic industry. The application of compact spectrometers enables the rapid measurement of samples while simultaneously reducing the consumption of resources and energy. Yet, this approach requires comprehensive data preprocessing and, on occasion, multivariate data analysis. For the process monitoring of the production of DHA, a low‐field ^1^H nuclear magnetic resonance (NMR) spectrometer was implemented in on‐line mode. Small‐volume samples were taken from a bypass and transferred to the spectrometer by an autosampler. Complete analysis within minutes allowed real‐time process control. To this purpose, reliable automated spectral preprocessing preceded the creation of a univariate model. The model enabled the acquisition of process knowledge from chemical kinetics and facilitated the tracking of both substrate and product concentrations, requiring independent calibration. As a second multivariate approach, principal component analysis was utilized to monitor the process in a semi‐quantitative manner without the necessity for calibration. The results of this study are beneficial for real‐time monitoring applications with the objective of exerting control over the process in question while minimizing expenditure.

## 1 Introduction

One approach to transforming the production of everyday products into sustainable processes is to utilize waste and by‐products as raw materials for new compounds, e.g. platform chemicals. Another way is to switch from chemical syntheses to biotechnological production processes (Anastas and Eghbali 2010; Horváth and Anastas 2007; C.‐J. Li and Trost 2008; Matthes and Schmid 2024; Stanek‐Wandzel et al. 2023). Process analytical technologies have grown into an essential element of energy and resource‐efficient production. Ideally, real‐time monitoring is desired to identify deviations from an expected process progress. Based on the real‐time data and process models, control measures can be taken to revert a deviating process into specifications. This will result in reduced product divergence, therefore less waste of resources, and reduced environmental impact (Armenta, Garrigues, and de la Guardia 2008; R. W. Kessler 2006; Legner et al. 2019; Munson, Freeman Stanfield, and Gujral 2006; Tobiszewski, Mechlińska, and Namieśnik 2010). Single quantity sensors such as pH, gas, and temperature sensors, deliver real‐time data, but with limited information content. In contrast, spectroscopic methods offer the advantage of multi‐component information while providing much faster measurement times than their chromatographic counterparts, e.g. high‐performance liquid chromatography (HPLC) (Bakeev 2010; R. W. Kessler 2006). Compact spectrometers, like NMR, near‐infrared (NIR), and Raman instruments, further combine energy‐efficient analyses with affordable procurement and operation. Their comparably small dimensions, low energy consumption and rather high robustness also allow for on‐site analysis. Yet, they often sacrifice spectral dispersion, resolution, and/or sensitivity (Beć, Grabska, and Huck 2021; Blümich 2019; Dalitz et al. 2012; Grootveld et al. 2019; Kreyenschulte et al. 2015; Leary, Crocombe, and Kammrath 2021; Meyer et al. 2016; Mitchell et al. 2014; van Beek 2021). These drawbacks can often be mitigated effectively by using appropriate data preprocessing and multivariate data analysis (Ge, Song, and Gao 2013; W. Kessler 2006; Rathore, Bhushan, and Hadpe 2011). As further desired features, automated analyses should be conducted on the raw medium, without the need for extended sample preparation, such as the addition of a deuterated solvent, which is often required for nuclear magnetic resonance (NMR) spectroscopy (Grootveld et al. 2019; Meyer et al. 2016; van Beek 2021). This can be achieved via flow cells, flow reactors, probes, or by using a bypass (De Beer et al. 2011; Giraudeau and Felpin 2018; Grootveld et al. 2019; Legner et al. 2019; Maschmeyer et al. 2020; Maschmeyer, Yunker, and Hein 2022).

As part of process monitoring with the aim of control, single‐point variables such as the pH value or the pressure can be kept at a constant level. An alternative approach would be to monitor their evolution over time. Since spectroscopic monitoring usually yields multicomponent information, it is often possible to trace educts, products, and—depending on the method's sensitivity—by‐products simultaneously. If the complex data can be simplified to a single variable that correlates with the concentrations of a calibration data set, univariate data analysis is straightforward, easy to interpret, and requires a minimum amount of computational power. In the case of highly complex data, such as crowded spectra, more sophisticated chemometric methods or multivariate data analysis techniques are typically required to simultaneously take advantage of all variables. Principal component analysis (PCA) is a widely used non‐regressional i.e., qualitative, multivariate data analysis technique, that is useful to identify similarities and differences between data series, such as spectra. While it is not suitable for the determination of concentrations or other quantitative variables, it can still be employed for process monitoring in a semi‐quantitative manner(Greenacre et al. 2022; De Ketelaere, Hubert, and Schmitt 2015; W. Li et al. 2000). Regressional methods of multivariate data analysis techniques, such as partial least squares regression (PLS‐R), are employed to derive concentrations from spectra, whereby the sections with the greatest amount of information are extracted and linked to the calibration (R. W. Kessler 2006; W. Kessler 2006).

In the cosmetics industry, glycerin, a by‐product of biodiesel production, is enzymatically oxidized to DHA in an aqueous medium by the acetic acid bacterium *Gluconobacter oxydans*(Hekmat, Bauer, and Fricke 2003; Liebminger et al. 2014; Pagliaro et al. 2007). The demand for this active ingredient in self‐tanning products is estimated to be up to 10,000 tons per year in the EU alone (ECHA—European Chemicals Agency 2024). To achieve the highest possible production rate, it is of the utmost importance to prevent the concentrations of both the substrate and the product from reaching toxic levels for the acetic acid bacteria and to ensure, that the activity of the bacteria does not decline (Claret, Bories, and Soucaille 1993; Stasiak‐Różańska, Błażejak, and Gientka 2014). However, it is nearly impossible to take corrective action in a timely manner using typical chromatographic analytical methods such as high‐performance liquid chromatography (HPLC), which is very time‐consuming and, in the case of glycerin and DHA, almost unable to separate the two components due to their molecular similarity. In comparison, NMR offers very good selectivity (Blümich 2019).

In this study, a low‐field compact ^1^H NMR spectrometer was applied for the real‐time monitoring of dihydroxyacetone production by *Gluconobacter oxydans*. The transformation of glycerin to DHA was conducted in a batch reactor. The spectrometer was coupled to an autosampler, enabling the automated collection of samples via a bypass. Off‐line analysis was conducted using HPLC as a reference. Appropriate pretreatment methods for the NMR‐based data were employed. The data were first analyzed using a univariate model. A concentration‐time (*c‐t*) diagram was established to gain process knowledge from chemical kinetics. Secondly, a semi‐quantitative analysis was performed using PCA. Based on the comparison of both methods, the efficacy of compact NMR spectrometers in combination with automated data analysis was demonstrated for the monitoring of the biocatalyzed conversion of glycerin to DHA.

## 2 Materials and Methods

### 2.1 Microorganisms

The *Gluconobacter oxydans* DSM 50049 strain was acquired from the Leibniz Institute DSMZ–German Collection of Microorganisms and Cell Cultures GmbH (Braunschweig, Germany) (Leibniz Institute DSMZ‐German Collection of Microorganisms and Cell Cultures GmbH 2023).

### 2.2 Cultivation

The medium for submerged cultivation of the microorganism was prepared according to Medium 360 of the Leibniz Institute DSMZ‐German Collection of Microorganisms and Cell Cultures GmbH (Braunschweig, Germany) (Leibniz Institute DSMZ‐German Collection of Microorganisms and Cell Cultures GmbH 2007), optimized according to Hu et. al.(Hu et al. 2010). It contained 40 g/L of mannitol (≥ 98%, Carl Roth GmbH + Co. KG, Karlsruhe, Germany), 4.5 g/L of glycerin (≥ 98%, Carl Roth GmbH + Co. KG, Karlsruhe, Germany), 5 g/L of yeast extract (Carl Roth GmbH + Co. KG, Karlsruhe, Germany), and 3 g/L of peptone from casein (Carl Roth GmbH + Co. KG, Karlsruhe, Germany). The medium was adjusted to pH 5 by adding 1 M hydrochloric acid (Merck KGaA, Darmstadt, Germany). The incubation process was carried out in a baffled Erlenmeyer flask (Duran, Carl Roth GmbH + Co., KG Karlsruhe, Germany) on a shaker at 140 rpm for 24 h at 26°C. Before use, each medium was previously autoclaved at 120°C for 20 min.

### 2.3 Production

After cultivating *Gluconobacter oxydans*, the cells were separated from the medium by centrifugation at 4500 G for 15 min at room temperature. They were then transferred to the production medium, according to Steinbüchel, which filled half of a 3‐liter bioreactor (Sartorius AG, Göttingen, Germany) containing 50 g/L of glycerin (≥ 98%, Carl Roth GmbH + Co. KG, Karlsruhe, Germany), 7.5 g/L of yeast extract (Carl Roth GmbH + Co. KG, Karlsruhe, Germany), 3 g/L of ammonium sulfate (≥ 99.5%, Carl Roth GmbH + Co. KG, Karlsruhe, Germany), and a solution of 1 M calcium chloride (Carl Roth GmbH + Co. KG, Karlsruhe, Germany) (Steinbüchel et al. 2012). The pH of the solution was adjusted to pH 5 using 1 M hydrochloric acid (Merck KGaA, Darmstadt, Germany). The bioreactor was stirred at 600 rpm and aerated at a rate of 1 L/min. To prevent foam formation and clogging of the gas outlet, 0.3 mL of Antifoam 204 (Sigma‐Aldrich Chemie GmbH, Taufkirchen, Germany) was added. The temperature was maintained at 27°C. After 25 h, 10 g/L glycerin was added to the bioreactor to test for an increase in dihydroxyacetone production. The process was terminated after 44 h.

### 2.4 Automated Sampling

**Figure 1:**
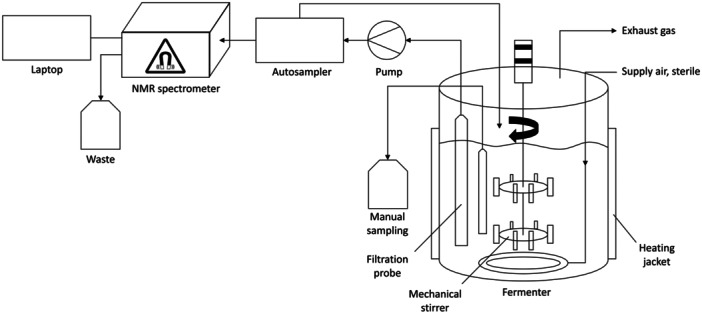
Diagram of the setup for automated sampling and NMR spectroscopic measurements of the biotechnological production of DHA by *Gluconobacter oxydans*.

To achieve fully automated sampling, an HPLC pump (KNAUER Wissenschaftliche Geräte GmbH, Berlin, Germany) set to 1 mL/min circulated the production medium continuously through a stainless‐steel flow cell connected to a MultiPurposeSampler 2 L (GERSTEL GmbH & Co. KG, Mülheim an der Ruhr, Germany) and returned it to the bioreactor, as illustrated in Figure 1. To prevent both clogging and the continuation of the production of DHA, the medium was filtered through a filtration probe equipped with a ceramic filter (TRACE Analytics GmbH, Braunschweig, Germany). Every 60 min, the autosampler transferred a sample of 400 µL of the medium from the flow cell through an injection valve to the picoSpin 80 ^1^H NMR spectrometer (Thermo Fisher Scientific GmbH, Dreieich, Germany). A JavaScript program embedded in the autosampler software initiated the measurement after a waiting time of 2 s after the injection, resulting in 45 spectra. Removal of the microorganisms by filtration ensured the absence of reaction progress during sample measurement. Spectroscopic analyses were performed in accordance with the methodology described below. Following the completion of each 5‐min sample measurement, the flow cell was rinsed with deionized water as part of the automation, thereby transferring the measured sample to the designated waste receptacle. Additionally, twelve samples of 1.5 mL each were manually collected at the outset, after 1, 2, 3, 21, 22, 23, 24, 25, 26, 27, and 44 h. They were centrifuged at 10,000 G for 10 min and subsequently frozen at −21°C for purposes of measurement via HPLC.

### 2.5 Spectral Recording and Processing

The NMR spectra were recorded online using a picoSpin 80 ^1^H NMR spectrometer (Thermo Fisher Scientific GmbH, Dreieich, Germany) operating at a proton Larmor frequency of 82 MHz. The permanent magnet temperature was kept at 36°C. The integrated flow cell with an inner diameter of 0.4 mm and an active volume of 40 nL allowed for rapid equilibration of the sample and the measurement of sheer liquid media without the necessity for further sample preparation or reference standards. The spectrometer was controlled using Thermo Fisher picoSpin software 1.0.1 (Thermo Fisher Scientific GmbH, Dreieich, Germany), accessed through the web browser Firefox (Mozilla Corporation, San Francisco, CA, USA) at the current version. A spectrum was recorded every 60 min, for which 32 scans were averaged. The pulse length was set to 58 µs, representing a pulse of 90°. A recovery delay of 500 µs preceded signal acquisition. The free induction decay was recorded as 4096 data points, followed by a relaxation delay of 8 s. Spectra were recorded from 3.2 to 5.1 ppm with a bandwidth of 4 kHz and preprocessed using MestReNova 12.0.0 (Mestrelab Research S.L., Santiago de Compostela, Spain) on a Windows 7 laptop computer. Following spectral phase correction, baseline correction with a third‐order Bernstein polynomial fit was performed. The spectra were then referenced to the water resonance at 4.7 ppm. Subsequently, all spectra were collected and saved in a CSV file format for further processing using the software MatLab R 2021a (MathWorks Inc., Natick, MA). The signals for glycerin and dihydroxyacetone were extracted between 3.62 and 3.68 ppm and between 4.40 and 4.4 ppm, respectively, and used in univariate models after the subsequent preprocessing. The methylene signal of glycerin at approximately 3.55 ppm was excluded from further analysis since no distinct changes could be observed during the reaction. The glycerin signal ranges were corrected for the offset, yielding the zero level for the intensity scale. The peak height, that is the maximum value of the glycerin peaks, was employed to construct a univariate model. Due to the proximity to the water resonance and subsequent decline in the baseline, first‐order derivation of the DHA signals was applied. The maximum intensity of a given signal represented hence the steepest slope of the dihydroxyacetone signal. The maximum values were correlated with the concentration values obtained from the chromatographic determination and were therefore used for the univariate and multivariate models.

### 2.6 Chromatographic Separation and Processing

The quantitative reference measurements of glycerin and dihydroxyacetone were conducted using the HPLC system Smartline (KNAUER Wissenschaftliche Geräte GmbH, Berlin, Germany), which was equipped with a Eurokat H column (KNAUER Wissenschaftliche Geräte GmbH, Berlin, Germany) with dimensions of 300 × 8 mm and a particle size of 10 µm. The isocratic eluents consisted of 90% double‐distilled water and 10% 50 mM sulfuric acid with a flow rate of 0.8 mL/min. Each chromatographic run lasted for 50 min. The system was equipped with a refractive index (RI) detector and an ultraviolet (UV) detector detecting at 200 nm. The column temperature was maintained at 40°C, while the autosampler was set to 10°C. Each injection consisted of 20 µL. The chromatograms were processed and evaluated using EuroChrom® 1.57 software (KNAUER Wissenschaftliche Geräte GmbH). For calibration, the co‐elution and hence the superposition of the two components during detection were taken into account. Dihydroxyacetone was detected by both the UV and the RI detectors, while glycerin was only observed by the RI detector. Equation 1 yielded the glycerin concentration *c(glycerin*_*sample*_ *)* from the proportion of dihydroxyacetone in the RI‐detected signal of the sample (*RI‐area*_*sample*_), from which was subtracted the peak area of both substances in the UV‐detected signal of the sample (*UV‐area*_*sample*_).

(1) $$c\left(\mathrm{glyceri}n_{\mathrm{sample}}\right)=\frac{\mathrm{RI}-\mathrm{are}a_{\mathrm{sample}}-\frac{\mathrm{UV}-\mathrm{are}a_{\mathrm{sample}}}{F}}{S}$$

with *S* being the slope of the linear calibration function for glycerin, and *F* being a dimensionless factor representing the proportion of dihydroxyacetone in the RI‐detected chromatograms, cf. equation 2.

(2) $$F=\frac{\mathrm{UV}-\mathrm{are}a_{\mathrm{DHA}}}{\mathrm{RI}-\mathrm{are}a_{\mathrm{DHA}}}$$

The quantity of dihydroxyacetone was readily determined by analyzing the peak area of the UV‐detected chromatograms.

### 2.7 Univariate Data Analysis

The concentrations of the substrate and the product were calculated through univariate analysis by correlating the results of the HPLC measurements with the corresponding preprocessed signal data. The glycerin concentrations of the samples taken at 2, 3, and 21 h needed to be excluded from analysis due to a temporary malfunction of the RI detector. Regression equations were then derived and used to calculate the concentrations of glycerin and dihydroxyacetone in each spectrum. The progress of both the substrate glycerin and the product dihydroxyacetone was then modeled using modified sigmoidal functions by Gompertz calculated by using the Curve Fitting Toolbox 3.5.13 of the software MatLab R 2021a (MathWorks Inc., Natick, MA) using Equations (3), (4), (5) (Zwietering et al. 1990).

(3) $$c_{\mathrm{glycerin},1}\left(t\right)=a_{1}-\left[A_{1}∙\text{exp}\left(-\text{exp}\left(\frac{{µ}_{m,1}∙e}{A_{1}}∙\left(\lambda -t\right)+1\right)\right)\right]$$

(4) $$c_{\mathrm{glycerin},2}\left(t\right)=a_{2}-\left[A_{2}∙\text{exp}\left(-\text{exp}\left(\frac{{µ}_{m,2}∙e}{A_{2}}∙\left(\lambda -\left(t-25\;h\right)\right)+1\right)\right)\right]$$

(5) $$c_{\mathrm{DHA}}\left(t\right)=A_{1}∙\text{exp}\left(-\text{exp}\left(\frac{{µ}_{m,1}∙e}{A_{1}}∙\left(\lambda_{1}-t\right)+1\right)\right)+A_{2}∙\text{exp}\left(-\text{exp}\left(\frac{{µ}_{m,2}∙e}{A_{2}}∙\left(\lambda_{2}-t\right)+1\right)\right)$$

where *t* represents the time in hours, *a* the calculated starting point in g/l, *A* the mean maximum value reached in g/L, *µ*_*m*_ the maximum specific growth rate in h^−1^ and *λ* the lag time in hours (Zwietering et al. 1990). As a result of adding the glycerin solution to the reactor after 25 h, two equations needed to be adjusted to model the glycerin concentration. For a one‐stage process, only Equation 3 and the first summand of Equation 5 are necessary.

### 2.8 Principal Component Analysis

Further to univariate data analysis, PCA was employed for the monitoring of the reaction progression, yielding semi‐quantitative diagrams and trends. The NMR spectra were preprocessed as described above. The two preprocessed segments, comprising 171 and 83 data points for glycerin and DHA, respectively, were subjected to two individual PCA for glycerin and DHA using PLS_Toolbox version 9.0 for MatLab (Eigenvector Research Inc., Manson, WA). The resulting scores were plotted along the first two principal components. Additionally, the scores of the first principal component were plotted against the reaction time.

## 3 Results and Discussion

### 3.1 Univariate Data Analysis

**Figure 2:**
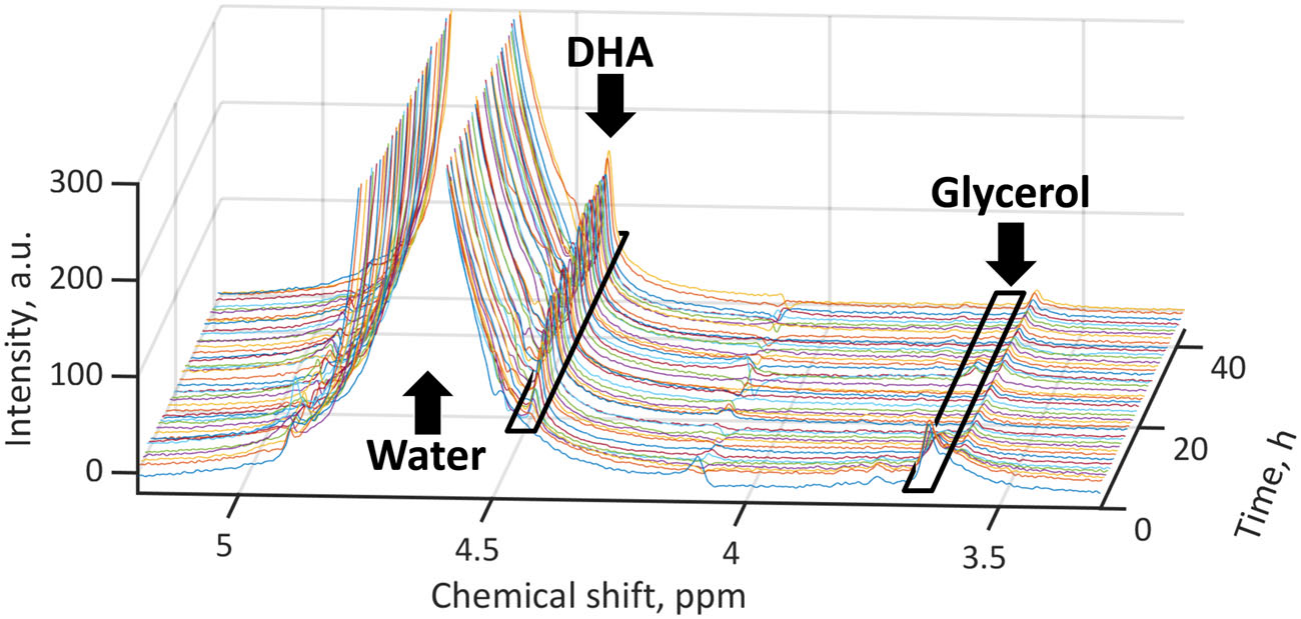
Low‐field ^1^H NMR spectra recorded during monitoring of the biotechnological transformation of glycerin to dihydroxyacetone. The spectra were referenced to the water signal at 4.7 ppm, baseline‐ and phase‐corrected, showing the substrate peak at 3.65 ppm and the product peak at 4.44 ppm together with the selected spectral ranges for the following analysis.

**Figure 3:**
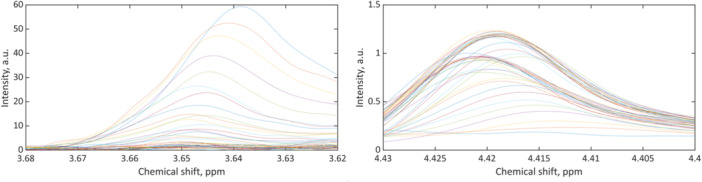
Preprocessed NMR spectra of glycerin (left) after spectral range selection and offset correction and of dihydroxyacetone (right) after forming the first derivative and spectral range selection. Note: Only the positive side of the derived signal is shown.

**Figure 4:**
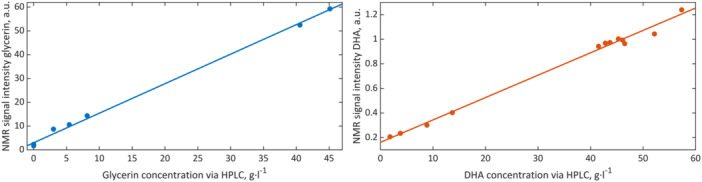
Calibration function of glycerin (left) and dihydroxyacetone (right). Signal intensities obtained from the preprocessed NMR spectra are plotted versus the concentrations obtained from the HPLC reference analysis. The corresponding parameters and adjusted coefficients of determination are listed in Table 1.

Combining the compact NMR spectrometer with an autosampler according to Figure 1 permitted sample processing and recording of NMR spectra in a fully automated manner. The short measurement times allowed for the attainment of sufficient temporal resolution to monitor the fermentation process in real‐time. The NMR spectra showed the predominant resonance of the water solvent at 4.7 ppm, as depicted in the spectra series shown in Figure 2. Database spectra and spectral prediction of glycerin and dihydroxyacetone revealed that the resonance at 3.65 ppm could be assigned to the substrate glycerin, while the product DHA was observed through its resonance at 4.44 ppm (Wishart et al. 2022). Although the glycerin signals and their fine structure were not fully resolved at 80 MHz magnetic field strength and both glycerin and DHA lacked the signal‐to‐noise ratio and sensitivity provided by high‐field spectrometers, they proved suitable for reaction monitoring purposes after careful preprocessing. For the purpose of quantification, the preprocessed data shown in Figure 3 were correlated with the results obtained from the HPLC reference data utilized in this study. The good linear correlation between NMR and HPLC methods is illustrated in Figure 4. The corresponding parameters and adjusted coefficients of determination, R²_adj._, are listed in Table 1. While for quantitation, integration of signals is usually the method of choice when applying NMR spectroscopy, the use of signal intensity provided better correlations in this study. After normalization, the peak height, i.e., maximum values of the glycerin peaks, was used to build a univariate model. Yet, the signals of DHA required extended processing due to their proximity to the water resonance and a subsequent declining baseline. This was accomplished through first‐order derivation and selection of the positive maximum, which is the higher field half of the signal. The maximum of each derived spectrum hence represented the steepest slope of the dihydroxyacetone signal. The thus processed signal exhibited the strongest correlation with the concentration values obtained from the chromatographic determination and was therefore used for the univariate model.

**Table 1:** Parameters of the linear calibration functions of glycerin and DHA based on the preprocessed NMR spectra.

Parameter	Glycerin	DHA
Slope^a^	1.241	0.01821
Intercept	2.996	0.1614
R^2^_adj._	0.997	0.993

^a^ l ∙ g^−1^

**Figure 5:**
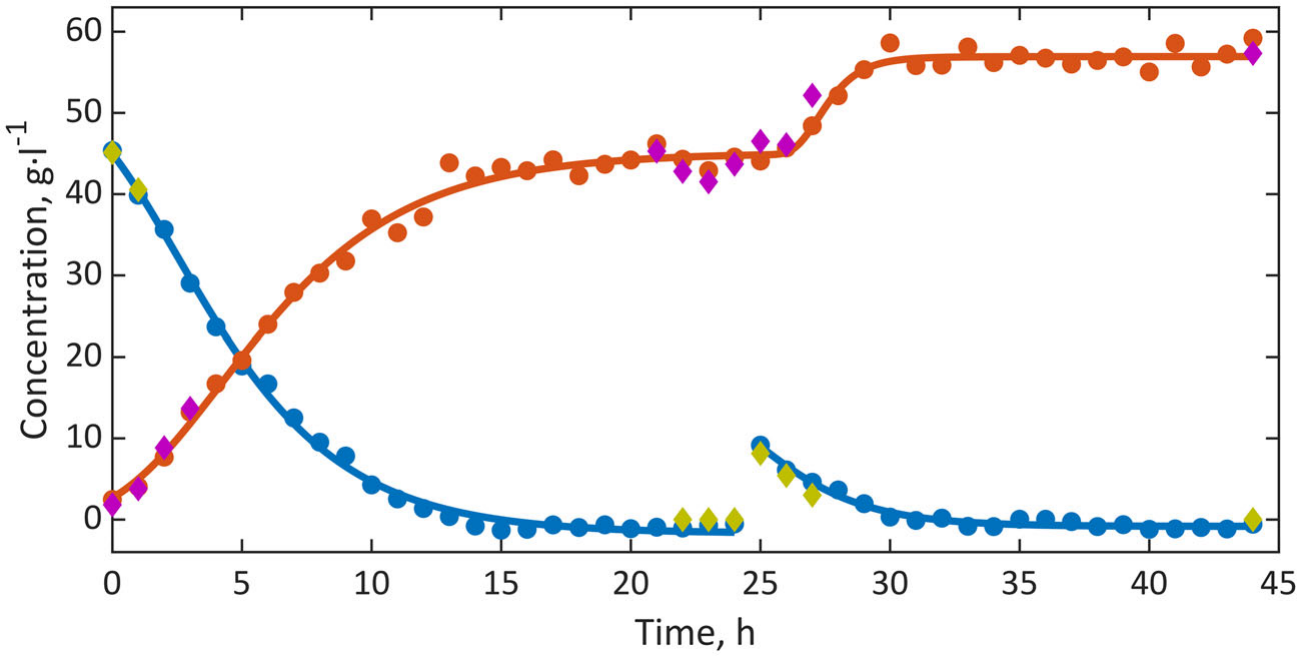
Concentration‐time diagram of the biotechnological DHA production process, based on the low‐field ^1^H NMR spectra. Calibration data of glycerin (yellow diamonds) and DHA (purple diamonds) based on the HPLC data are plotted along with the concentrations of glycerin (blue circles) and DHA (orange circles) determined by univariate data analysis based on the NMR spectra together with their fits (lines) according to equations (3), (4), (5).

Substrate and product preprocessed spectral series yielded excellent calibrations for both glycerin and dihydroxyacetone, as indicated by the coefficients of determination close to 1 from the linear regressions in the NMR‐HPLC diagram. The high precision was mainly achieved by selecting the spectral ranges as shown in Figure 3, where the broad water resonance and the noise from other regions were excluded. The model was then used to determine the concentrations of the process constituents in samples during the conversion. The results were graphed as a concentration‐time diagram, cf. Figure 5. This diagram represents the time course of substrate and product concentrations, with the concentration data of the HPLC reference analysis, presented as diamonds and the concentrations calculated from the NMR spectra presented as circles. The typical progression of product formation in biotechnological fed‐batch processes can be recognized. During the initial hours, bacterial growth led to an acceleration in DHA production, as indicated by the steep rise of the growth function. The production was then inhibited by the remaining substrate in the reactor, causing the process to decelerate. After approximately 15 h, all glycerin was consumed, resulting in a constant product concentration of around 43 g/L. Following a second addition of glycerin at 25 h in the form of a 10 g/L solution to the batch, the production of dihydroxyacetone resumed. It is important to note that the non‐steady course of the substrate function is the consequence of the second glycerin addition. The resulting final concentration of approximately 57 g/L of DHA represented a yield of nearly 97%.

The glycerin and DHA concentrations obtained from the univariate method were computed according to the Gompertz equations (3), (4), (5) by mathematical optimization using the fit parameters listed in Table 2. The high degree of consensus observed in the fits indicates a highly accurate description of the process.

**Table 2:** Fit parameters of the univariate models for predicting the glycerin and dihydroxyacetone concentrations using Gompertz equations.

Parameter	DHA	Glycerin
a_1_ ^a^	—	52.43
a_2_ ^a^	—	14.84
A_1_ ^a^	45.08	54.13
A_2_ ^a^	11.86	15.67
µ_m,1_ b	4.14	5.499
µ_m,2_ b	4.85	2.452
λ_1_ c	0.15	−1.144
λ_2_ c	26.31	−2.384

^a^ g ∙ l^−1^,

^b^ h^−1^,

^c^ h.

As illustrated, A_1_ and A_2_ represent the mean maximum values of the dihydroxyacetone concentrations before and their increase after the addition of the 10 g/L glycerin solution, respectively. The similarity of the maximum specific growth rates *µ*_*m*,1_ and *µ*_*m*,2_ indicates that the bacteria retained their ability to convert glycerin to DHA during the 10‐h non‐productive period without substrate. The process can hence be halted and restarted as needed. The process monitoring also proved sufficiently robust to yield consistent data after process resumption.

The univariate model graphed as a *c‐t* diagram could be employed to monitor and control the fermentation process. The values obtained during monitoring can be utilized to identify the point at which the substrate has been depleted. Subsequently, the approach is well‐suited for automated substrate supply and for implementing countermeasures in the event of deviations from the standard process. As the product has a toxic effect on the acetic acid bacteria at higher concentrations, the model indicates the optimal time to halt the process, add substrate, or remove reactants. Furthermore, the NMR spectroscopic monitoring is sufficiently accurate to indicate the fitness of the bacteria for repetitive feed.

### 3.2 Principal Component Analysis

**Figure 6:**
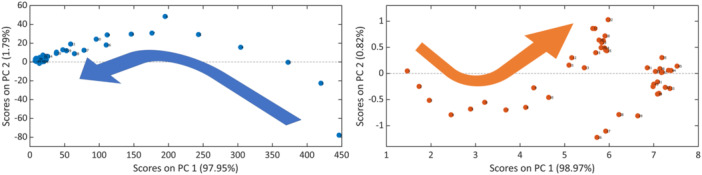
Scores plots of the PCAs of glycerin (left) and DHA (right): Second versus first principal components with the direction of the process (arrow).

To illustrate its utility for process monitoring in a semi‐quantitative manner, without the necessity for calibration, the selected preprocessed spectral regions were subjected to PCA, resulting in the typical score plots. Both reaction constituents reflected the course of the reaction, cf. Figure 6. Product and substrate show, as desired, opposite directions along with the biochemical transformation progress. Analysis of the score plots revealed that the main information for the process status was contained along PC 1. However, the score plot of the DHA monitoring demonstrated the addition of substrate as a disruption along PC 2, followed by the continuation of the reaction along PC 1. The two steady states, i.e., before and after the addition of substrate, were represented as clusters in the scores, due to the absence of further changes. As PCA reveals variations in the data, no quantitative information is contained. Since the biochemical reaction causes a steady decrease or increase of reaction components, the variation can be interpreted as a trend such that in a sequence of data increase and decrease along a principal component can be interpreted as an increase or decrease in concentration.

**Figure 7:**
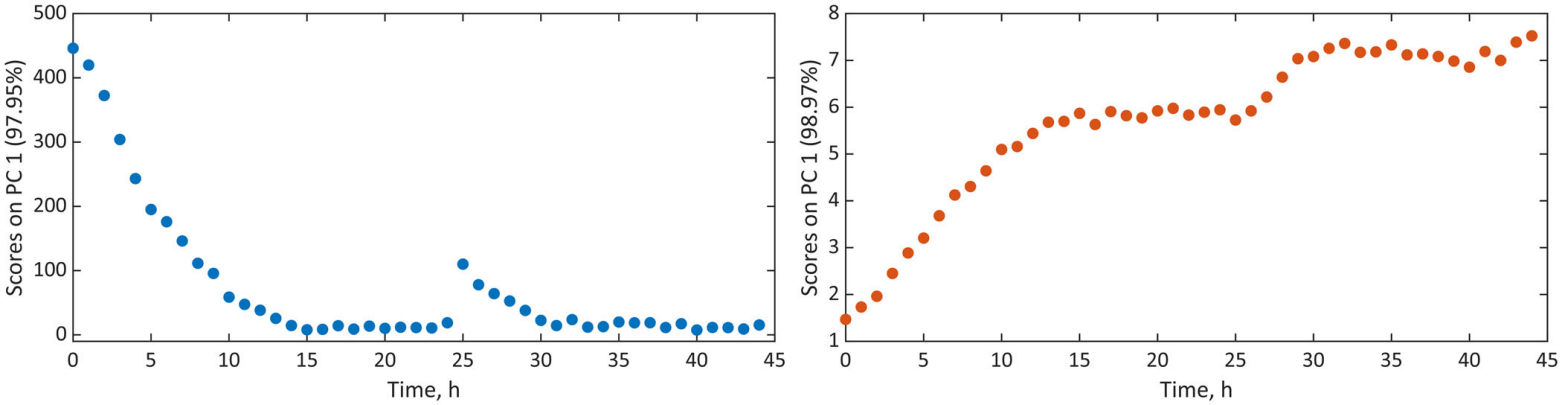
PCAs of glycerin (left) and dihydroxyacetone (right): first principal component (PC1) scores versus the reaction time.

Moreover, while reaction time is not explicitly included in PCA, it may be reintroduced when sampling occurred at known intervals and the order of samples is considered. As stated above, relative concentrations may also be derived along with the process progress. While Figure 7 does not reflect concentrations directly, both scores plots are highly comparable to the *c‐t* representations of both glycerin and dihydroxyacetone from univariate data analysis and calibration as shown in Figure 5. Since the sampling time was known, the data could be associated with the reaction time. Consequently, PC1 versus time yielded the equivalent of semiquantitative *c‐t* diagrams. They appear hence suitable for reaction monitoring. As data preprocessing and multivariate data analysis by PCA can be performed in an automated manner and in a relatively short time, the approach can be used to identify deviations in a well‐known process, as well as univariate methods. It is further useful for cases where calibrations are highly time‐consuming and knowledge of precise concentrations is not relevant or cannot be gained. The univariate approach, when combined with appropriate preprocessing, enabled the accurate quantitation of substrate and product. It further provided the description according to chemical or biochemical kinetics and would hence be applicable to prevent the occurrence of toxic levels of both substrate and product for the bacteria. A high production rate can thus be maintained. In contrast, PCA and its representations would be useful for non‐calibrated monitoring purposes. Further to the conventional PCA representation, the plotting of the scores of the first principal component against the reaction time provided an accessible and interpretable way to describe the process semi‐quantitatively.

## 4 Conclusion

The combination of a low‐field compact ^1^H NMR spectrometer with an autosampler‐controlled bypass and appropriate data pretreatment methods proved suitable for real‐time monitoring the biotechnological production of dihydroxyacetone from glycerin by *Gluconobacter oxydans*. The low resolution and sensitivity of compact spectrometers were successfully overcome by preprocessing the relevant signals and utilizing calibration, univariate and multivariate analysis, leading to quantitative and semi‐quantitative concentration‐time diagrams suitable as models for reaction monitoring or control. Moreover, the models were capable of describing a successive addition of glycerin to result in a multi‐step process. A maximum yield of dihydroxyacetone of 97% was found. Both approaches may be used for process control to prevent both substrate and product concentrations from reaching toxic levels for the bacteria. Additionally, it was shown that the bacteria retained their conversion ability even after periods of glycerin absence. To further enhance the yield, high‐performance yeast strains may be employed in conjunction with automated strategies for substrate addition and product segregation. The utilization of low‐field ^1^H‐NMR spectroscopy to monitor bioprocesses may assist in the reduction of analytical waste and energy expenditure through the analysis of a minimal number of small‐volume samples. Furthermore, this approach could be amended to on‐site analysis.

## Author Contributions

**Lukas Mahler:** writing–review and editing, writing–original draft, visualization, validation, methodology, investigation, formal analysis, data curation, conceptualization. **Ebru Tasdemir:** validation, methodology, investigation. **Anna Nickisch‐Hartfiel:** writing–review and editing, supervision, resources. **Christian Mayer:** writing–review and editing, supervision. **Martin Jaeger:** writing–review and editing, supervision, resources, formal analysis, conceptualization.

## Conflicts of Interest

The authors declare no conflicts of interest.

## Data Availability

The data that support the findings of this study are available from the corresponding author upon reasonable request.
